# The influence of COVID-19-related resilience on depression, job stress, sleep quality, and burnout among intensive care unit nurses

**DOI:** 10.3389/fpsyg.2023.1168243

**Published:** 2023-05-02

**Authors:** Sojin Hwang, Jungmin Lee

**Affiliations:** ^1^Department of Clinical Nursing Science, Hallym University, Chuncheon-si, Gangwon-do, Republic of Korea; ^2^School of Nursing, Hallym University, Chuncheon-si, Gangwon-do, Republic of Korea

**Keywords:** burnout, COVID-19, nurse, resilience, job satisfaction

## Abstract

The COVID-19 pandemic has placed immense pressure on healthcare workers, in particular, Intensive Care Unit (ICU) nurses, who are at the forefront of managing critically ill COVID-19 patients. This has led to increased stressors and workload, which are associated with negative mental health outcomes such as depression, job stress, sleep disturbances, and burnout. However, COVID-19-related resilience may have mitigated these negative effects. ICU nurses with higher levels of COVID-19-related resilience may be better equipped to manage the stress and job demands during the pandemic, leading to improved mental health outcomes. Therefore, this study aimed to comprehensively explore the factors influencing the resilience of ICU nurses and provide baseline knowledge for future studies to develop interventions that promote COVID-19-related resilience. With shift work and COVID-19 experience with adult patients from hospitals across three regions of South Korea. The questionnaire included scales/measures of nurses’ depression, job stress, sleep quality, and burnout. Results confirmed that resilience was negatively correlated with depression and burnout, and that ICU nurses’ relative levels of resilience strongly influenced their experience of burnout. The findings of this study make a significant contribution to the literature because they focus on resilience, specifically in the context of ICU nursing in South Korea, which has become more challenging and demanding due to the pandemic.

## 1. Introduction

In the context of the prolonged COVID-19 pandemic, medical staff continue to provide frontline patient care in hospitals. Among them are nurses, who play a key role in limiting the spread of the pandemic (Fawaz et al., 2020). Nurses take on additional tasks, such as infection-prevention education for patients, guardians, and caregivers; monitoring the symptoms of patients and caregivers; infection control; caregiver management; and record keeping (Chen et al., 2020). Additionally, their workload has increased since the advent of the pandemic due to additional infection-control tasks, such as managing isolation rooms and wearing protective gear (Bernard et al., 2009; Corley et al., 2010).

COVID-19 has significantly impacted healthcare workers, particularly those working in intensive care units (ICUs) (Chen et al., 2020). High-flow nasal cannulas, mechanical ventilation, extracorporeal membrane oxygenation (ECMO), continuous renal replacement therapy, prone position, and nursing interventions are required for severely ill patients infected with COVID-19 (The Korean Society of Critical Care Medicine, 2020). Accordingly, the government has designated a hospital dedicated to infectious diseases, which provides a negative pressure isolation room to treat patients with COVID-19 (The Korean Society of Critical Care Medicine, 2020).

The COVID-19 pandemic has placed immense pressure on healthcare workers, particularly those working in ICUs. Consequently, ICU nurses may experience a range of negative psychological and physical outcomes, including depression, job stress, poor sleep quality, and burnout (Nie et al., 2020; Sun et al., 2020). Their physical and mental stress and fatigue accumulate due to additional tasks such as cleaning and arranging the ICU environment, resulting in severe mental distress and lack of sleep (Zhuo et al., 2020). An overseas study reported that nurses who took care of patients with COVID-19 wore personal protective equipment for hours at a time, resulting in pressure sores on the body; some nurses experienced hypoglycemia and oxygen deprivation due to physical fatigue (Buheji and Buhaid, 2020). Nurses experienced facial pain, had communication difficulties because they could not hear properly, and felt severe fatigue because of problems such as excessive work when wearing personal protective equipment while working (Maben and Bridges, 2020).

ICU nurses have been at the forefront of managing critically ill COVID-19 patients, which has led to increased work demands and stressors. However, COVID-19-related resilience might play a significant role in mitigating these negative effects. Resilience is an individual’s ability to successfully adapt to adversity (Eoh et al., 2019). Long-term exposure to the ICU environment can lead to poor sleep quality, stress, and depression, all of which can affect nurses’ resilience. Resilience is a key factor that can help ICU nurses cope with stressors associated with the COVID-19 pandemic. Resilience refers to an individual’s ability to adapt and recover from adversity or stress. Resilient ICU nurses may be better equipped to cope with the challenges of their jobs, may experience fewer negative outcomes and have improved mental health outcomes (Cooper et al., 2020). Previous studies have reported that nurses with low resilience lack the ability to deal with stress, resulting in negative consequences such as burnout or job turnover (Kang and Lim, 2015; Ryu and Kim, 2016; Kwon et al., 2017). Conversely, highly resilient nurses can overcome difficulties and demonstrate improved abilities by actively utilizing their internal and external resources (Kwon et al., 2017). Highly resilient nurses also had lower turnover intention (Kim and Park, 2010). However, not everyone experiences emotional pain from traumatic experiences, and those who experience trauma can overcome it with resilience (Tusaie and Dyer, 2004; Eoh et al., 2019).

According to a meta-analysis of factors affecting resilience among Korean nurses, empathy satisfaction, nursing performance, job satisfaction, organizational socialization, social support, and organizational commitment are protective factors that have a positive effect on resilience (Kwon et al., 2017). In contrast, stress response, burnout, turnover intention, workplace bullying, empathic fatigue, job stress, and post-traumatic stress disorder are risk factors that negatively affect resilience (Kwon et al., 2017). There are limited studies focusing on the resilience of ICU nurses during the COVID-19 pandemic, including general characteristics, job characteristics, depression, job stress, sleep quality, and burnout. Therefore, research that comprehensively explores factors that influence ICU nurses’ resilience is needed.

Overall, COVID-19-related resilience may have significantly influenced the mental health outcomes of ICU nurses during the pandemic. Nurses who possess higher levels of resilience may be better equipped to manage the stress and demands of their job, leading to improved mental health outcomes, such as reduced depression, job stress, sleep disturbances, and burnout. It is essential to develop interventions that promote COVID-19-related resilience among ICU nurses to mitigate the negative impact of the pandemic on their mental health. Thus, in this study, depression, job stress, sleep quality, and burnout levels were assessed in ICU nurses who had cared for patients with COVID-19. The impact of these factors was identified and analyzed to promote resilience among nurses. This study aimed to provide basic data for increasing the competency of nursing personnel and the quality of their services by improving the quality of their professional lives and work intentions.

## 2. Materials and methods

### 2.1. Purpose

This descriptive study investigated the factors that influence ICU nurses’ resilience during COVID-19, specifically in terms of depression, job stress, sleep quality, and burnout. This study intends to provide basic data for improving the quality of life and work intention of nursing personnel by promoting resilience among ICU nurses.

### 2.2. Participants

Convenience sampling was used for the recruitment. This study was conducted among ICU nurses working at different university hospitals in three regions across South Korea. Written consent for voluntary participation was obtained after explaining that participants could withdraw from the study without facing any disadvantages. The inclusion criteria were as follows: (1) ICU nurses with shiftwork experience and (2) nurses who nursed patients with COVID-19 in adult ICUs. G*Power 3.1.9.4 was used to calculate the minimum sample size required for linear multiple regression analysis to identify the factors influencing resilience. Based on a significance level (α) of 0.05, power of 0.95, effect size of 0.15, and four predictors (depression, job stress, sleep quality, and burnout), the minimum sample size was calculated as 129 for the analysis. Considering a dropout rate of 20%, 155 questionnaires were distributed. Finally, 131 (84.5%) questionnaires were used for the analysis; questionnaires with missing responses were excluded.

### 2.3. Instruments

#### 2.3.1. Depression

The Center for Epidemiologic Studies Depression Scale (CES-D) developed by Radloff (1977) was adapted and validated, and the integrated Korean version of the scale developed by Chon et al. (2001) was used to measure depression. This tool consists of 20 items rated on a scale ranging from 0 (“not at all”) to 3 (“very much so”). A score of 21 indicates mild depression, and a score of 21 or higher indicates severe depression (0–15 indicates no depression, 16–20 indicates mild depression, and 21 and above indicates severe depression). Cronbach’s α in the study by Chon et al. (2001) and in this study were 0.91 and 0.92, respectively.

#### 2.3.2. Job stress

A questionnaire developed and used by Oh (2016) to investigate MERS-related stress among medical staff in 2015 and modified by Song and Yang (2021) was used to measure COVID-19-related job stress. Among the stress emotions, the questionnaire included six items on fear (items 2, 3, 4, 7, 8, and 11), four on alienation (items 1, 6, 9, and 10), and one each on betrayal (item 5) and anger (item 12). Each item is scored on a 5-point Likert scale (1 = “not at all,” 2 = “no,” 3 = “normally,” 4 = “yes,” and 5 = “very much”). Cronbach’s α in Song and Yang’s (2021) study and in this study were 0.88 and 0.90, respectively.

#### 2.3.3. Sleep quality

The Pittsburgh Sleep Quality Index (PSQI) developed by Buysse et al. (1989) is the basis of the Korean version (PSQI-K) translated by Sohn et al. (2012). The PSQI-K measures the subjective sleep quality over the past month and consists of seven domains: subjective sleep quality, sleep latency, sleep duration, usual sleep efficiency, sleep disturbance, use of sleeping pills, and daytime dysfunction. It consisted of 19 items. A score of 0—3 was assigned to each domain, with a total score ranging from 0 to 21 points. Higher scores indicate poorer sleep quality, and a score of 5 or more indicates poor sleep. The Cronbach’s α in the study by Buysse et al. (1989) and in this study was 0.83 and 0.65, respectively.

#### 2.3.4. Burnout

Burnout was measured using the Korean version of the Maslach Burnout Inventory (MBI) (Shin, 2003) developed by Maslach and Jackson (1981). This 22-item tool consists of three sub-domains: dehumanization (five items), low self-achievement (eight items), and emotional exhaustion (nine items). On a 7-point Likert scale, higher scores indicated higher levels of burnout. Items on low self-achievement, which were positive statements, were reverse-scored. Cronbach’s α in the study by Maslach and Jackson (1981) and in this study were 0.76 and 0.85, respectively.

#### 2.3.5. Resilience

As a tool for measuring resilience, the Conner-Davidson Resilience Scale (CD-RISC) developed by Connor and Davidson (2003) was adapted and validated by Baek et al. (2010) and the Korean Version of the scale was used in this study. At the time of development, the tool consisted of 25 items with five sub-factors: tenacity (nine items), persistence (eight items), optimism (four items), sustaining power (two items), and spirituality (two items). The items are rated on a 5-point scale ranging from 0 (not at all) to 4 (very much); the total scores range from 0 to 100, with higher scores indicating higher levels of resilience. Cronbach’s α was 0.90 in the study by Connor and Davidson (2003) and was 0.95 in this study.

### 2.4. Data collection

This study was approved by the institutional review board of H University (HIRB-2022-055). The study period was from August 12 to September 12, 2022. The researcher personally visited the departments and distributed envelopes containing an explanation of the study, consent form for participation, and questionnaire. Considering that the training period for new nurses in the ICU was 6 weeks, the target sample consisted of nurses with more than 2 months of ICU experience who understood the content and responded to the questionnaire. The questionnaire required 20–30 min to complete.

### 2.5. Data analysis

Statistical data, such as descriptive statistics, independent *t*-test, one-way ANOVA, Pearson’s correlation coefficients, and multiple linear regressions, were analyzed using SPSS WIN 29.0.

## 3. Results

### 3.1. General characteristics, including participants’ and COVID-19-related job characteristics

The average age of the participants was 28.71 (±5.49) years; most (68.7%, *n* = 90) were in their 20s, more than 90% (*n* = 118) were women, and 67.2% (*n* = 88) did not subscribe to any religion (Table 1). The highest educational level was an undergraduate degree at 83.2% (*n* = 109), and 84.7% (*n* = 111) were unmarried. Regarding the number of cohabitants, 40.5% (*n* = 53) lived alone, 21.4% (*n* = 28) lived with one other person, 21.4% (*n* = 28) lived in households with four or more people, and 16.8% (*n* = 22) lived in three-person households. Of the nurses who lived with others, 68.7% (*n* = 90) lived with their immediate families.

**Table 1:** General characteristics of the participants (*N* = 131).

	Categories	*N* (%)	M ± SD (min-max)
General characteristics			
Age (years)	<30	90 (68.7)	28.71 ± 5.49 (22–52)
	≥30	41 (31.3)	
Sex	Male	13 (9.9)	
	Female	118 (90.1)	
Religion	Yes	43 (32.8)	
	No	88 (67.2)	
Educational level	Community college degree	7 (5.3)	
	Bachelor’s degree	109 (83.2)	
	Attending Gradate school or master’s degree	15 (11.5)	
Marital status	Unmarried	111 (84.7)	
	Married	20 (15.3)	
Number of households	Alone	53 (40.5)	
	Two	28 (21.4)	
	Three	22 (16.8)	
	More than four	28 (21.4)	
Residence type	Immediate family	90 (68.7)	
	Extended family	41 (31.3)	
Job related characteristics			
Total clinical experience (month)	≤36	55 (42.0)	65.15 ± 63.62 (4–365)
	37 ∼ 60	24 (18.3)	
	61 ∼ 120	34 (26.0)	
	>120	18 (13.7)	
Clinical experience at the ICU (month)	≤36	66 (50.4)	52.66 ± 47.97 (4–252)
	37 ∼ 60	24 (18.3)	
	61 ∼ 120	28 (21.4)	
	>120	13 (9.9)	
Current department experience (month)	≤36	77 (58.8)	42.37 ± 41.10 (1–240)
	37 ∼ 60	25 (19.1)	
	61 ∼ 120	23 (17.6)	
	>120	6 (4.5)	
Types of ICU	Medicine	40 (30.5)	
	Surgical	40 (30.5)	
	Cardiovascular	17 (13.0)	
	Nervous System	6 (4.6)	
	Smart ICU	14 (10.7)	
	Emergency ICU	14 (10.7)	
Assigned to the current workplace	Volunteer	77 (58.8)	
	From the hospital	54 (41.2)	
Position	Registered nurse	126 (96.2)	
	Charge nurse or higher	5 (3.8)	
Satisfaction with current workplace	Dissatisfied	9 (6.9)	
	Neutral	59 (45.0)	
	Satisfied	63 (48.1)	
Satisfaction with workload at current workplace	Dissatisfied	30 (22.9)	
	Neutral	74 (56.5)	
	Satisfied	27 (20.6)	
Number of patients	<3	26 (19.8)	2.98 ± 0.74 (1–6)
	3	92 (70.3)	
	>3	13 (9.9)	
Turnover experience	Yes	34 (26.0)	
	No	97 (74.0)	
Work characteristics related to COVID-19			
Workload Work intensity (Difficulty)	Same as before	20 (5.3)	
	Increased	84 (64.1)	
	Greatly increased	27 (20.6)	
	Same as before	29 (22.2)	
	Increased	78 (59.5)	
	Greatly increased	24 (18.3)	
Salary satisfaction	High	11 (8.4)	
	Low	120 (91.6)	
Experience of receiving education for infected patients	Yes	68 (51.9)	
	No	63 (48.1)	
Availability of resources	Have	43 (32.8)	
	Not have	88 (67.2)	
Quarantined experience due to infection	Yes	98 (74.8)	
	No	33 (25.2)	

The average total clinical experience of nurses was 65.15 (±63.62) months; 42.0% (*n* = 55) had less than 36 months, followed by 26.0% (*n* = 34) with 61–120 months. Among them, nurses who were currently working in the ICU had an average of 42.37 (±41.10) months of clinical experience and 58.8% (*n* = 77) had 36 months or less of ICU experience. By department, 30.5% (*n* = 40) were medical ICU (MICU) nurses and 30.5% (*n* = 40) worked in the surgical ICU (SICU), followed by 15.3%. in the smart ICU and nervous system ICU, 13.0% (*n* = 17) in the cardiovascular ICU (CCU), and 10.7% (*n* = 14) in the emergency ICU.

Of the ICU nurses in this study, 58.8% (*n* = 77) desired to be assigned to their current workplace; however, 41.2% (*n* = 54) did not. A majority were general nurses (96.2%, *n* = 126) and less than half were very satisfied or satisfied with their work (48.1%, *n* = 63). This was followed by 45.0% (*n* = 59) who had neutral feelings about it. Regarding nurses’ satisfaction with the workload in the current workplace, 56.5% (*n* = 74) answered neutral, 22.9% (*n* = 30) were very dissatisfied or dissatisfied, and 20.6% (*n* = 27) were very satisfied or satisfied. The average number of patients assigned to the nurses was 2.98 (±0.74), with more than 70% (*n* = 92) having three patients, 19.8% (*n* = 26) having less than three, and 9.9% (*n* = 13) having four or more. Of the nurses, 74.0% (*n* = 97) had never changed their workplace and 26.0% (*n* = 34) responded that they had experienced switching jobs.

Regarding COVID-19-related work, 64.1% (*n* = 84) answered that there was an increase in workload during the pandemic, 18.3% (*n* = 27) reported a sharp increase in their duties, and 13.0% (*n* = 17) reported having the same workload as before. Approximately 60% (*n* = 78) responded that their work intensity had increased, and over 90% (*n* = 120) were dissatisfied with the benefits or rewards they received during COVID-19 nursing care. Slightly over half (51.9%, *n* = 68) of the nurses had received education on responding to infectious diseases. A total of 67.2% (*n* = 88) had not received sufficient resources during the COVID-19 pandemic, and 74.8% (*n* = 98) responded that they had quarantine experience due to COVID-19.

### 3.2. Degree of depression, job stress, sleep quality, burnout, and resilience

Participants’ average depression score was 15.50 (±9.02) out of a total possible score of 60; 55.7% (*n* = 73) of participants had no depression, 25.2% (*n* = 33) had severe depression, and 19.1% (*n* = 25) had mild depression (Table 2).

**Table 2:** The level of depression, job stress, sleep quality, burnout, and resilience of the participants (*N* = 131).

Variables	Categories	*N* (%)	Mean ± SD
Depression	Normal (≤15)	73 (55.7)	15.50 ± 9.02
	Mild (16∼20)	25 (19.1)	
	Moderate (≥21)	33 (25.2)	
Job stress	Fear		2.85 ± 0.76
	Isolation		2.24 ± 0.82
	Betrayal		2.15 ± 1.07
	Anger		2.08 ± 1.12
	Sub-total		2.52 ± 0.73
Sleep quality (*n* = 129)	GOOD (<5)	51 (38.9)	5.29 ± 2.56
	BAD (≥5)	78 (59.5)	
Burnout	Emotional exhaustion		2.71 ± 1.23
	Decreased of self-fulfillment		2.74 ± 1.16
	Dehumanization		2.11 ± 1.31
	Sub-total		2.78 ± 0.80
Resilience	Toughness		2.20 ± 0.62
	Persistence		2.31 ± 0.64
	Optimism		2.23 ± 0.73
	Bearing capacity		3.00 ± 0.69
	Spirituality		1.81 ± 0.69
	Sub-total		2.27 ± 0.56

Participants’ COVID-19-related work stress had an average score of 2.52 (±0.73) out of a maximum of 5. In the sub-categories, fear had the highest score at 2.85 (±0.76), followed by alienation at 2.24 (±0.82), betrayal at 2.15 (±1.07), and anger at 2.08 (±1.12) points.

The average score for sleep quality was 5.29 (±2.56) out of a total score of 21. Approximately 60% (*n* = 78) had poor sleep quality, and two-fifths (38.9%, *n* = 51) had good sleep quality.

Participants’ average score for burnout was 61.16 (±17.57) points out of a total score of 132. Emotional exhaustion had the highest score at 24.39 (±11.09), followed by a decreased sense of self-achievement at 21.89 (±9.26), and dehumanization at 10.56 (±6.53) points. According to the 27-point cutoff score, approximately six out of ten (58.0%, *n* = 7) had a low burnout level and 42.0% (*n* = 55) had a high burnout level.

Their average resilience score was 56.72 (±14.07) points out of a total score of 100. In the sub-categories, toughness had the highest score of 19.76 (±5.58), followed by 18.44 (±5.09) points for persistence, 8.91 (±2.90) for optimism, 6.00 (±1.38) for supportiveness, and 3.61 (±1.37) for spirituality.

### 3.3. Differences in the resilience according to the general characteristics of the participants

There were significant differences in resilience by participants’ gender (*t* = 2.053, *p* = 0.042), religion (*t* = 2.596, *p* = 0.011), educational level (*F* = 3.906, *p* = 0.023), position (*t* = 2.347, *p* = 0.020), and past experience in COVID-19 response training (*t* = 2.660, *p* = 0.009) (Table 3).

**Table 3:** Differences in resilience according to general characteristics (*N* = 131).

	Categories	Resilience	
		Mean ± SD	*t* or *F* (*p*) Scheffe’s test
Gender	Male	2.57 ± 0.43	2.053^*^ (0.042)
	Female	2.24 ± 0.57	
Religion	Yes	2.45 ± 0.50	2.596^*^ (0.011)
	No	2.18 ± 0.58	
Educational level	Community college degree	2.21 ± 0.62^a^	3.906^*^ (0.023) b < c
	Bachelor’s degree	2.22 ± 0.53^b^	
	Attending Gradate school or master’s degree	2.64 ± 0.68^c^	
Position	Registered nurse	2.24 ± 0.55	2.347^*^ (0.020)
	Charge nurse or higher	2.79 ± 0.72	
Experience of receiving education for infected patients	Yes	2.39 ± 0.56	2.660^**^ (0.009)
	No	2.14 ± 0.54	

*p < 0.05, **p < 0.01.

Male nurses had higher resilience levels than female nurses (2.57 ± 0.43 and 2.24 ± 0.57, respectively). In terms of religiousness, those who subscribed to a religion (2.45 ± 0.50) had higher resilience levels than those who did not (2.18 ± 0.58). Regarding educational level, those who were enrolled in or completed graduate school (2.64 ± 0.68) had significantly higher resilience than those who had associate or bachelor’s degrees (2.21 ± 0.62 and 2.22 ± 0.53, respectively). General registered nurses (2.24 ± 0.55) had lower resilience levels than charge nurses (2.79 ± 0.72). Lastly, participants who received COVID-19 response training (2.39 ± 0.56) had higher resilience levels than those who did not receive any related education (2.14 ± 0.54).

### 3.4. Correlations between factors affecting resilience

Resilience was significantly and negatively correlated with depression (*r* = −0.352, *p* < 0.001) and burnout (*r* = −0.510, *p* < 0.001) (Table 4). Hence, the lower the degree of resilience, the more severe the degree of depression and burnout.

**Table 4:** Correlation between depression, job stress, sleep quality, burnout, and resilience of participants (*N* = 131).

	Depression (Fawaz et al., 2020) *r* (*p*)	Job stress (Chen et al., 2020) *r* (*p*)	Sleep quality (Corley et al., 2010) *r* (*p*)	Burnout (Bernard et al., 2009) *r* (*p*)	Resilience (The Korean Society of Critical Care Medicine, 2020) *r* (*p*)
1					
2	0.505^***^ (<0.001)				
3	0.394^***^ (<0.001)	0.236^**^ (0.007)			
4	0.667^***^ (<0.001)	0.578^***^ (<0.001)	0.307^***^ (<0.001)		
5	−0.352^***^ (<0.001)	−0.113 (0.200)	−0.165 (0.062)	−0.510^***^ (<0.001)	

**p < 0.01, ***p < 0.001.

### 3.5. Predictors of participants’ resilience

Analysis found that the tolerance limit was greater than 0.10, and the variance inflation factor to confirm multicollinearity between independent variables was 1.802 or less than 10, indicating that there was no problem with multicollinearity (Table 5). In addition, the Durbin-Watson value was 2.008, indicating no autocorrelation between the independent variables. The analysis showed that burnout had a statistically significant effect on resilience (*β* = −0.496, *p* < 0.001), but the effect of depression was insignificant (*β* = −0.021, *p* = 0.840). That is, the goodness-of-fit of the regression model was statistically significant for burnout level and showed an explanatory power of 26.1% %in the multiple regression analyses on resilience. These findings indicate that when nurses’ burnout levels decreased, the predicted resilience level increased.

**Table 5:** Factors affecting resilience (*N* = 131).

Variables	*B*	S.E	*β*	*T*	*p*	VIF
	81.53	3.967		20.551	<0.001	
Depression	−0.032	0.159	−0.021	−0.202	0.840	1.802
Burnout	−0.398	0.082	−0.496	−4.866	<0.001	1.802

*R*^2^ = 0.510 Adju-*R*^2^ = 0.261, *F* = 22.55, *p* < 0.001, Durbin-Watson 2.008.

## 4. Discussion

This study assessed depression, job stress, and sleep quality levels of nurses in ICUs who had taken care of patients with COVID-19 and analyzed the impact on resilience to improve nurses’ resilience and the quality of their professional lives. This study aimed to improve the quality of nursing services by increasing the quality of nursing personnel, their competency, and their intention to stay in their position at work.

In this study, the average depression score of the participants was 15.50 (±9.02) out of 60; 58 nurses (44.3%) had depression scores of 16 or higher, indicating that they had mild or high levels of depression. According to a study by Gong and Kim (2022) the depression level of nurses who took care of critically ill patients at university hospitals was relatively higher than that of nurses who took care of mildly ill patients at medical centers. Therefore, during the COVID-19 pandemic, it is necessary to monitor, intervene, and manage depression in ICU nurses, who have a relatively high psychological burden.

In this study, participants’ COVID-19-related occupational stress score was an average of 2.52 (±0.73) out of five points. Using the same job-stress tool as this study to investigate nurses at infectious disease hospitals, Song and Yang (2021) and Kim (2020) reported scores of 3.05 (±0.67) and 3.09 (±0.67) points, respectively, which are higher than those of this study. Among them, as a subdomain of COVID-19-related job stress, the average value of the fear domain was the highest at 2.85 (±0.76) points. This was similar to Song and Yang (2021) and Kim’s (2020) study of infectious disease hospital nurses and Cho’s (2021) study of emergency room nurses, in which the mean value of the fear domain was the highest. These results indicate that anxiety about being directly or indirectly exposed to infection while nursing patients with COVID-19 and concerns about risk factors that may occur while doing so acted as stressors.

In this study, the average sleep quality score was 5.29 (±2.56) out of a total score of 21, indicating a low sleep quality. Good sleep quality supports optimal immune function to prevent infection during direct contact with patients (Connor and Davidson, 2003). Poor sleep quality may lead to medical and safety accidents that could harm nurses’ health and affect their work performance (Baek et al., 2010). According to previous studies on sleep deprivation in nurses, deterioration of sleep quality due to shift work among nurses is not only related to individual health problems such as activation of the sympathetic nervous system, increased susceptibility to infection, cognitive impairment, emotional changes, and physical pain, but also nurse turnover (Hong, 2015; Jeong and Gu, 2016; Yang et al., 2017; Yeong and Ock, 2018). Poor sleep quality has also been reported to reduce the accuracy and efficiency of nurses’ work, thereby increasing the risk of errors in medication administration, patient identification, medical device operation, and needle injury, affecting patients’ health and life (Hong, 2015; Jeong and Gu, 2016; Yang et al., 2017; Yeong and Ock, 2018).

The average burnout score in this study was 61.16 (±17.57) points out of 132 points. This result is higher than the average score (56.90 ± 20.20) reported by Shin (2003) who measured burnout in ICU nurses who cared for critically ill patients with COVID-19. This study may be different depending on the timing of the study in that it was conducted right after the surge in COVID-19 patients. For nurses, burnout causes frequent absenteeism and increases the possibility of medical accidents and turnover rates (Eo, 2015). Therefore, support measures should be developed to manage burnout among ICU nurses. Regarding the subcategories of burnout, emotional exhaustion was the highest (24.39 points), followed by decreased self-achievement (21.89 points) and dehumanization (10.56 points). This result is consistent with those of previous studies (Shin, 2003; Chen et al., 2020; Kim, 2020).

In this study, the average resilience score was 2.27 (±0.56) out of the 5. This was lower than Cho (2021) (3.28 points) and Yang et al. (2017) (3.30 points), who studied nurses in hospitals dedicated to COVID-19 using the same tool. In addition, Jeong and Gu (2016) who studied ICU nurses’ resilience during non-epidemic periods, reported scores of 2.43 points, which is higher than the results of this study. This suggests that the resilience of nurses in ICUs decreases when dealing with infectious diseases. Therefore, the resilience of ICU nurses who nursed critically ill COVID-19 patients was lower. Social support systems such as mentoring or counseling are needed to improve resilience.

There was a significant difference in the degree of resilience according to participants’ gender, religion, educational level, position, and experience of receiving education about the COVID-19 response. According to a study by Woo (2022) during the pandemic, resilience was significantly higher when nurses had a high educational level and rank, which is similar to the results of this study. Similarly, Lee (2022) found that resilience was significantly higher when the nurse in charge was older, religious, and held a higher position. In other words, resilience increased when work experience increased. Therefore, it is necessary to create an environment that promotes nurses’ retention by developing and implementing programs to increase resilience. Additionally, resilience was high among those who received education on how to respond to infectious diseases, suggesting that nurses should be provided with information on how to respond to infectious diseases.

Regarding COVID-19, ICU nurses’ resilience negatively correlated with depression and burnout. The more severe the degree of depression and the higher the burnout, the lower is the resilience. A previous study reported that the higher the depression, the lower the resilience of shift nurses (Jeong and Young, 2019) and another study of ICU nurses showed that the lower the resilience, the higher the depression (Mealer et al., 2012). In addition, previous studies reported that the higher the resilience of nurses at public medical centers who care for patients with COVID-19, the lower their burnout (Yeong and Ock, 2018). Nurses with low resilience have a low ability to effectively control or manage stress, so burnout and turnover are high (Tusaie and Dyer, 2004). Therefore, it is necessary to consider measures to reduce ICU nurses’ burnout in future infectious disease situations and develop interventions to enhance resilience. Further research is needed to fully contextualize the situation in South Korea and compare it with other countries.

## 5. Contributions and limitations

This study provides basic data for interventions that promote COVID-19-related resilience among ICU nurses and aim to mitigate the negative impact of the pandemic on their mental health. The findings revealed that ICU nurses with high levels of depression and burnout experienced low resilience. This was significantly affected by burnout. Therefore, it is necessary to develop and implement support measures to manage burnout among ICU nurses and promote their resilience. Furthermore, the study found that the resilience of ICU nurses decreased depending on general characteristics such as gender, religion, education level, position, and experience of receiving education for infected patients. Social support systems, such as mentoring or counseling that consider individuals’ general characteristics, may be beneficial in improving the resilience of ICU nurses.

This study has some limitations that should be considered when interpreting the results. First, it used convenience sampling and had a relatively small sample size compared with previous research on clinical nurses, which limits its generalizability to a broader population in South Korea. Therefore, future research should use a larger and more diverse sample to confirm whether these findings replicate with more representative South Korean populations. Second, the study focused mainly on nurses currently working in university hospitals, which limits the applicability of the findings to other groups. Nurses employed in diverse hospitals should also be surveyed to draw definitive conclusions regarding ICU nurses’ resilience. Third, longitudinal studies are needed to identify other significant predictors of resilience toward infectious diseases. Future research should also consider other sociodemographic variables beyond country, age, and sex, which may contribute to non-invariant results. Finally, the potential for self-report errors was considered. Although self-reported measures are commonly used in research, they may not always provide a complete or accurate picture of an individual’s experiences or behaviors. Thus, future study is needed including an alternative research methodology that could address this concerns such as qualitative and/or mixed-method.

## Data availability statement

The raw data supporting the conclusions of this article will be made available by the authors, without undue reservation.

## Ethics statement

The studies involving human participants were reviewed and approved by Hallym University’s institutional review board (HIRB-2022-055). The patients/participants provided their written informed consent to participate in this study.

## Author contributions

SJH and JML contributed in conception and design, provision of study materials or participants, data collection and intervention implementation, data analysis and interpretation, manuscript writing, and revision of the manuscript. All authors have read and agreed to the published version of the manuscript.

## Conflict of interest

The authors declare that the research was conducted in the absence of any commercial or financial relationships that could be construed as a potential conflict of interest.

## Publisher’s note

All claims expressed in this article are solely those of the authors and do not necessarily represent those of their affiliated organizations, or those of the publisher, the editors and the reviewers. Any product that may be evaluated in this article, or claim that may be made by its manufacturer, is not guaranteed or endorsed by the publisher.
